# Improvement in Methane Production from Pelagic *Sargassum* Using Combined Pretreatments

**DOI:** 10.3390/life12081214

**Published:** 2022-08-10

**Authors:** Karla Daniela Chikani-Cabrera, Patricia Machado Bueno Fernandes, Raúl Tapia-Tussell, David Leonardo Parra-Ortiz, Galdy Hernández-Zárate, Ruby Valdez-Ojeda, Liliana Alzate-Gaviria

**Affiliations:** 1 Renewable Energy Unit, Yucatan Center for Scientific Research, Carretera Sierra Papacal-Chuburná Puerto, Km 5, Sierra Papacal, Mérida 97302, Mexico; 2 Center for Biotechnology, Federal University of Espírito Santo—UFES, Campus Maruípe, Vitória 29043900, Espírito Santo, Brazil; 3 Colegio de Postgraduados, Campus Veracruz, Posgrado en Agroecosistemas Tropicales, Mpio. Manlio Fabio Altamirano, Veracruz 91700, Mexico

**Keywords:** anaerobic digestion, pelagic *Sargassum*, pretreatments, direct interspecies electron transfer

## Abstract

The constant golden tides of *Sargassum* spp., identified to be a mixture of *Sargassum natans* and *Sargassum fluitans*, observed recently in the Mexican Caribbean have affected the marine ecosystem and the local economy and have created the need for solutions for their management and use. The *Sargassum* arrivals have thus been considered as third-generation feedstock for biofuel. Their potential for energetic conversion to biomethane was investigated, with hydrolysis as the limiting step due to its complex composition; therefore, in the present study, different physical, chemical, and enzymatic pretreatments and a combination of them have been evaluated, with the additional use of granular activated carbon, to determine the best yield and methane quality. The combined pretreatments of 2.5% hydrogen peroxide, followed by an enzymatic pretreatment (enzymatic extract from *Trametes hirsuta* isolated from decomposing wood in the Yucatán Peninsula-Mexico), was the best option, reaching a biodegradability of 95% and maximum methane yield of 387 ± 3.09 L CH_4_/kg volatile solid. The use of a conductive material, such as granular activated carbon, did not generate significant changes in performance and methane concentration.

## 1. Introduction

There are approximately 350 species of the genus *Sargassum*. Two of these holopelagic *Sargassum* species, *S. natans* and *S. fluitans*, are the major contributor to golden tides [1]. The first golden tides appeared in 2011 in the North Atlantic; during the following years, they spread from West Africa to the Caribbean Sea and Gulf of Mexico [2]. The recurrent golden tides have caused damage to the marine ecosystem, causing the death of fish, damage to coral reefs, preventing the nesting of turtles, and causing coastal dead zones [3,4]. In 2015, 10,000 tons of *Sargassum* were removed from the beach daily [1]. Although the influx of *Sargassum* decreased significantly during 2016 and 2017, in 2018 and 2019 the phenomenon was repeated, so it is expected that the arrivals will persist [5].

A solution for the disposal of *Sargassum* is the production of biogas, with hydrolysis as the rate-limiting step due to its composition: insoluble fibers (7.15–75%) [6,7], lignin (15.6% to 29.5% dry basis) [8,9], heavy metals, salts (15.2–23.1% dry basis) [10], polyphenols (25.4% dry basis) [8], and a low beneficial ratio of carbon and nitrogen (less than 20:1) [10,11]. Pretreatments break down structures that are difficult for hydrolytic microorganisms to digest [9,12]. For this reason, physical pretreatments such as maceration reduce the size of the particles, increasing the surface area that allows sugars to be released [12]. Generally, the physical pretreatments carried out on different macroalgae have positive results, with yields from 92 L CH_4_/kg VS to 422 L CH_4_/kg VS [13,14,15,16,17,18].

The use of chemical pretreatments, such as acids or alkalis, changes the structural solid fractions which facilitate the biodegradability of the biomass. In alkali pretreatment, the biomass simultaneously undergoes solvation and saponification, degrading the lignin and cellulose components, thereby increasing the concentration of sugars accessible for microbial digestion. Comparatively, the use of acids is more effective than alkalis at accelerating hemicellulose depolymerisation and delignifying biomass [19]. Yields of 148 to 365 L CH_4_/kg VS have been reported for various macroalgae when using alkaline pretreatments such as NaOH [20,21,22], while for an acid pretreatment (HCl), the yields were from 52 to 312 L CH_4_/kg VS [20,21,22]; however, severe pretreatments, such as the use of chemicals, can release inhibitors such as furfural, organic acids, and phenolic compounds and alter the pH, inhibiting anaerobic digestion [23]. 

The biological pretreatment applied in several macroalgae, including the use of enzymes such as alginate lyases, laminarinases, or glucanases, for the degradation of polysaccharides in brown algae, were obtained from marine microorganisms [24,25,26] or seaweed composts [26]. The phenolic compounds and lignin [8] commonly found in *Sargassum* spp. can be oxidized by laccases [8,9]. Many white rot fungi, including *Trametes versicolor*, have been used for the selective degradation of lignin and hemicellulose. These fungi produce extracellular lignolytic enzymes such as lignin peroxidase, manganese peroxidase, and laccase [27,28]. The fungal pretreatment from *Trametes hirsuta* has increased the methane production for a pelagic *Sargassum* consortium from the Mexican Caribbean by 17% [9]. The use of enzymes as pretreatments results in yields from 49 L CH_4_/kg VS (*Fuculus vessiculosos*) [14] to 232 L CH_4_/kg VS (*Laminaria digitata* and *Saccharina latissima*) [29]. The use of extracellular enzymes is attractive due to their low cost, their extraction does not imply the use of chemicals that harm the environment, and they can be used as a pretreatment to improve the biodegradability of macroalgae [9].

Likewise, author Saratale et al., 2018, discuss in their review that the co-digestion of algal biomass with other waste substrates such as straws, waste paper, switch grass, glycerol, beet silage, and sewage sludge, among others, improve the C:N ratio which results in increased methane production [30].

A way to overcome the biochemical fermentative barrier in the process of increasing the production and quality of methane during anaerobic digestion is to promote the direct transfer of electrons between microorganisms (for example, *Geobacter metallireducens* and *Methanosaeta harundinacea*) from conductive materials such as granular activated carbon (GAC-low cost), biochar, nano-magnetite, and minerals [31,32]. Electrically conductive materials could substitute pilus and/or cytochromes to exchange electrons [33]. However, Valero et al. determined that the adsorption or promotion of DIET, when a conductive material was used, depended on the characteristics of the substrate; in the case of pot ale whiskey, the use of activated carbon did not increase performance in anaerobic digestion due to the adsorption of organic matter [34]. Shanmugam et al. used the GAC to adsorb inhibitors such as phenolic compounds and the concentration of COD before the anaerobic digestion begins, and, similar to other authors, the concentration of methane was mainly due to the characteristics of the substrate and the adsorption properties of the conductive material [35]. 

Therefore, the main objective of this work was to evaluate the potential of biogas production from pelagic *Sargassum* in the Mexican Caribbean, choosing the best pretreatment, whether physical, chemical, enzymatic, and/or combined, with and without granular activated carbon (DIET), that allows for improving production and quality methane. The use of hydrogen peroxide at low concentrations (2.5% *v*/*v*) has not yet been evaluated, similar to pretreatments in the production of methane from pelagic *Sargassum* spp.

## 2. Materials and Methods

### 2.1. Sample Collection 

Sample mixes of *Sargassum natans* and *Sargassum fluitans* (*Sargassum* spp.) were manually collected offshore in Puerto Morelos, Quintana Roo (2050.9195° N, 08652.5743° W), Mexico (2020 and 2021) during the autumn. The samples were washed superficially with tap water several times to remove impurities such as salts and sands and then dried at 80 °C (APHA 2005). The *Sargassum* spp. was stored in a cold room (4 °C) for their subsequent compositional analysis (moisture, ash, total solids, and volatile solids) and later use. 

### 2.2. Enzymes

The enzymes were obtained from the methodology reported by Tapia-Tussell et al. [9] which consisted of a strain of *T. hirsuta* Bm-2 (GQ280373) that was isolated from decaying wood in Yucatán, Mexico. The strain was maintained by periodical subculturing on plates with 2% (*w*/*v*) malt extract and 2% bacteriological agar (*w*/*v*). The plates were incubated at 35 °C for 4 or 5 days. Subsequently, a mycelia suspension of *T. hirsuta* was obtained by inoculating four 1-cm diameter plugs in a 250 mL flask previously sterilized at 121 °C for 15 min in a culture medium whose composition was (g/L): glucose 10 g, malt extract 10 g, peptone 2 g, yeast extract 2 g, KH_2_PO_4_ 2 g, hepta-hydrated MgSO_4_ 1 g, thiamine 1 g, and 2% (*w*/*v*) wheat bran. The fungus was incubated at 35 °C for 7 days at 150 rpm and subsequently filtered.

### 2.3. Inoculum

The inoculum consisted of a native mixed microbial consortium containing: 300 g/L bovine manure, 150 g/L pig manure, 30 g/L deep soil, and 1.5 g/L Na_2_CO_3_ [36].

### 2.4. Sample Characterization

Elemental analysis was determined using a Thermo Scientific Flash 2000 CHNS/O elemental organic analyzer and Thermo Scientific Flash 2000 software: EAGER Xperience version 1.2, Thermo Fisher Scientific, Waltham, MA, USA. From the results obtained from the elemental composition, the theoretical biochemical potential of methane from *Sargassum* spp. was determined according to Equations (1) and (2) reported by Ward et al. [37]. In Equation (1), the stoichiometric reaction of methane production, *a*, *b*, *c*, and *d* correspond to the molar composition of carbon, hydrogen, oxygen, and nitrogen.

(1) $$(C_{a}H_{b}O_{c}N_{d})+\left(\frac{4a-b-2c+3d}{4}\right)H_{2}O\to \left(\frac{4a+b-2c-3d}{8}\right)CH_{4}+\left(\frac{4a-b+2c+3d}{8}\right)CO_{2}+dNH_{3}$$

The theoretical methane yield can then be predicted by Equation (2) (L CH_4_/kg VS).

(2) $$\frac{L\;de\;CH_{4}}{kg\;SV}=\left(\frac{4a+b-2c-3d}{12a+b+16c+14d}\right)V_{m}$$

### 2.5. Biochemical Methane Potential (BMP) Test

The BMP tests followed protocols and calculations according to Valero et al. [38]. After the pretreatment of *Sargassum* ssp., BMP tests were carried out in triplicate in 250 mL serum bottles capped with rubber septum sleeve stoppers, with a useful volume of 140 mL and a headspace volume of 110 mL. The tests were carried out for 30 days at 40 °C; all tests were manually shaken once a day. Before the BMP test, the inoculum was degassed for 5 to 10 days. The inoculum/substrate ratio was 2 g VS inoculum/g VS *Sargassum* spp. and the bottles were filled with distilled water to complement the volume to 140 mL. In the same way, granular activated carbon (GAC) was added (to some pre-treatments) at a concentration of 40 g/L for microorganisms to exchange electrons with and promote an increase in methane gas production. To avoid the presence of oxygen, each of the reactors underwent oxygen displacement with nitrogen flux in an anoxic chamber. Three blanks with inoculum were also tested to measure its potential for methane production. Accumulated methane gas production was measured for the blanks and each of the tests to perform the pertinent calculations according to the methodology described by Valero et al. [38]. 

### 2.6. Physical Pretreatment

The samples were washed several times with tap water and then dried at 80 °C. Subsequently, mechanical cutting was performed with a blade blender (waring commercial, Blender 51BL30) and sieved until particle sizes greater than 1 mm were obtained.

### 2.7. Chemical Pretreatment

The chemical pretreatment consisted of 10 g of *Sargassum* spp. physically treated with 200 mL of 2.5% (*v*/*v*) hydrogen peroxide solution. The pretreatment was performed in triplicate.

### 2.8. Enzymatic Pretreatment

The enzymatic pretreatment, conducted in triplicate, consisted of a suspension of *Sargassum* physically treated with 10% *w*/*v* buffer solution to maintain the pH balance at 5. The buffer solution consisted of a 40% citric acid solution (0.05 M) and 60% sodium citrate solution (0.05 M), to this suspension, the enzyme extract with initial laccase activity of 7000 U/ML for each g of *Sargassum* was added, according to the report by Tapia-Tussell and collaborators [9]. The resulting suspension was incubated at 40 °C, 150 rpm for 48 h. 

### 2.9. Combined Pretreatments

Different combined pretreatments were performed in triplicate; chemical and enzymatic pretreatment (PE), which consisted of the previously physically treated *Sargassum* spp., followed by chemical and enzymatic pretreatment, and finally the PTE treatment which consisted of the previously physically treated *Sargassum* spp., then a chemical, thermal (120 °C in an autoclave for 15 min), and enzymatic pretreatment.

### 2.10. Scanning Electron Microscopy (SEM)

The effect of pretreatment was observed with a scanning electron microscope (SEM, model JSM-6360LV, JEOL, Tokyo, Japan). Four samples were mounted on a metallic stub using double-sided adhesive tape coated with a 15 nm gold layer and observed at 20 kV.

### 2.11. Statistical Analysis

The effect of various pretreatments was evaluated by Tukey’s post-hoc test following one-way ANOVA at *p*-values lower than 5%. All values are presented as average ± standard deviation. Excel 2019 (Microsoft Office ProPlus 365 64 bit) was used. The effect of pretreatment was observed with a scanning electron microscope (SEM, model JSM-6360LV, JEOL, Tokyo, Japan). Flour samples were mounted on a metallic stub using double-sided adhesive tape coated with a 15 nm gold layer and observed at 20 kV.

## 3. Results

### 3.1. Compositional Analysis

The *Sargassum* spp. collected in Puerto Morelos were analyzed by the moisture content, ash, total solids (TS), and volatile solids (VS) (Table 1); the values correspond to the two periods described in Section 2.1. 

#### CHNS/O

The results of the CHNS analysis (Table 2), are indicated. Based on CHNS/O, the empirical formula (normalized) can be expressed as CH_1.66_O_0.81_N_0.03_.

### 3.2. Biochemical Methane Potential (BMP)

#### 3.2.1. Methane Yield

**Figure 1:**
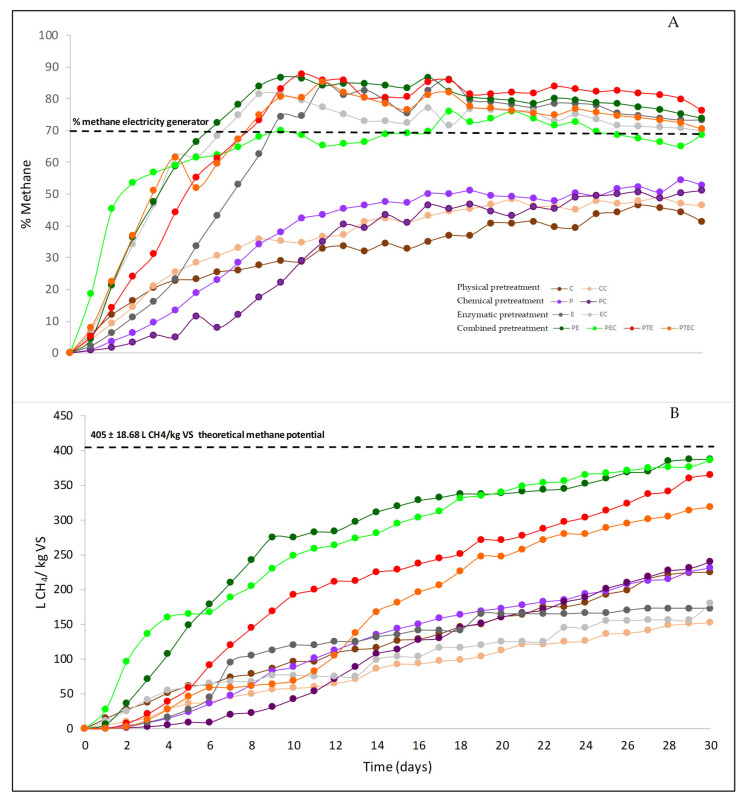
(**A**) Concentration of methane; (**B**) Methane yield accumulation. C and CC: physical pretreatment; P and PC: chemical pretreatment (peroxide 2.5%); E and EC: enzymatic pretreatment; PE and PEC: combined pretreatment chemical and enzymatic; PTE and PTEC: combined chemical, thermal, and enzymatic pretreatment. C means pretreatment with GAC.

The physical pretreatments evaluated with and without GAC (C and CC) (Figure 1A) rendered a methane accumulation of 224.19 ± 9.45 L CH_4_/kg VS and 152.89 ± 2.00 L CH_4_/kg VS, respectively. The chemical pretreatment (P and PC) resulted in a methane accumulation of 230.82 ± 11.65 L CH_4_/kg VS and 240.32 ± 3.04 L CH_4_/kg VS. The enzymatic pretreatments (E and EC) resulted in an accumulation of methane of 172.57 ± 0.56 L CH_4_/kg VS and 179.56 ± 0.50 L CH_4_/kg VS. The combined pretreatment of PEC, PTE, and PTEC rendered a methane accumulation of 385.73 ± 4.76 L CH_4_/kg VS; 364.95 ± 8.18 L CH_4_/kg VS; and 318.06 ± 10.24 L CH_4_/kg VS, respectively. The PE pretreatment (chemical and enzymatic treatment) resulted in a higher methane accumulation of 387.64 ± 1.41 L CH_4_/kg VS.

#### 3.2.2. Concentration of Methane

During the 30 days of experimentation, the methane concentration, shown in Figure 1B), and the maximum accumulation of the methane test were observed after the ninth and twelfth day. The physical pretreatments C and CC presented a methane concentration of 46.44% ± 1.69 and 48.86% ± 3.41, respectively. The chemical pretreatments P and PC resulted in methane concentrations of 51.55% ± 3.77 and 51.11% ± 0.56. In the pretreatments E, EC, ET, and ETC the concentration of methane was 84.56% ± 0.15; 81.49% ± 1.02; 84.56% ± 0.1; and 88.60% ± 4.57, respectively. Finally, with the combined pretreatments PE, PEC, PTE, and PTEC, the methane concentration was 86.41% ± 0.04; 76.04% ± 0.8; 87.69% ± 1.49; and 85.26% ± 0.1, respectively.

#### 3.2.3. Biodegradability Index

The biodegradability index (Table 3) results from dividing the biochemical potential of methane by the theoretical potential of methane. The combined pretreatment PE and PEC have a higher biodegradability index of 0.95, followed by the PTE pretreatment of 0.88.

### 3.3. Scanning Electron Microscopy (SEM)

**Figure 2:**
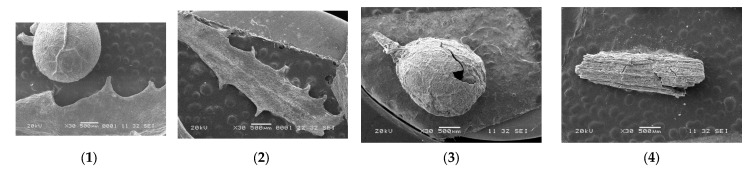

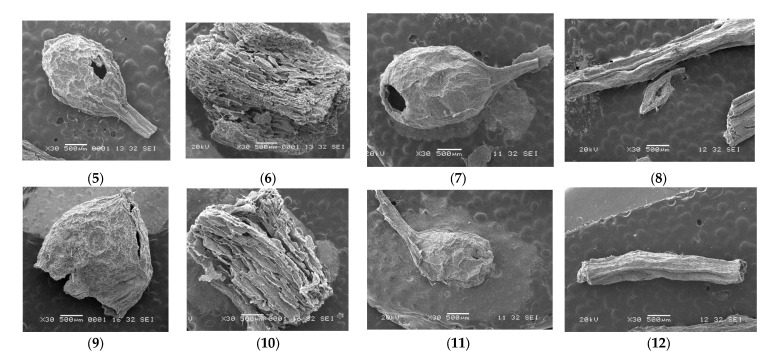
SEM: air vesicles without pretreatment (**1**) and lanceolate leaves without pretreatment (**2**); C: physical pretreatment (**3**) and (**4**); P: chemical pretreatment (**5**) and (**6**); E: enzymatic pretreatment (**7**) and (**8**); PE: combined pretreatment (chemical and enzymatic) (**9**) and (**10**); and PTE: combined pretreatment (chemical, thermal and enzymatic) (**11**) and (**12**).

The SEM results (Figure 2) of the air vesicles (1) that allow *Sargassum* spp. to float and the lanceolate leaves (2) without any previous pretreatment, indicated a completely smooth structure. After the physical pretreatment C, the air vesicle (3) has a hole in the upper part and its structure no longer looks as smooth; the same happens with the lanceolate leaves (4) which show cracks. The chemical pretreatments (P) with hydrogen peroxide provoke a higher number of cracks in the air vesicle (5) and lanceolate leaves of *Sargassum* spp. (6), indicating the treatment with 2.5% peroxide allowed the degradation of the *Sargassum* spp. structure. The enzymatic pretreatment (E), images (7) and (8) indicate there is no change in the structure of the *Sargassum* spp. For the combined PE pretreatments, images (9) and (10) indicate structural changes in the pelagic *Sargassum* can be observed; the same occurs with the combined PTE pretreatment images (11) and (12).

## 4. Discussion

### 4.1. Composition of Sargassum spp.

*Sargassum* ssp. collected in the Mexican Caribbean (autumn 2020 and 2021) contained 12.98% moisture after being dried at 80 °C for 24 h. This value is comparable to other *Sargassum* spp. (13.05% moisture) [39]. The pelagic *Sargassum* collected in Barbados (June 2018) resulted in 20.63% moisture after the *Sargassum* spp. was dried at 80 °C [11]. Generally, brown macroalgae such as *Sargassum* are high in moisture (80–90%) when they are not subjected to a drying process [10,12]. The ash content of *Sargassum* spp. collected in the Mexican Caribbean was 22.22%. Varied results have been found in the bibliography, for example, Oyesiku and Egunyomi found that *Sargassum* spp. collected in Nigeria contain 9.5% ash [7], while Morrison and Gray determined that *Sargassum* collected in the Caribbean contains 24% s asash [15], similar to this study. The ash content of the pelagic *Sargassum* reported by other authors was 31.82% to 46.94% (dry basis) [10,11]. These differences are mainly because the composition of the *Sargassum* varies considerably from place to place, season to season, and species to species [40]. Furthermore, the chemical composition, growth, and pigmentation of the *Sargassum* are significantly affected by conditions such as light, temperature, salinity, available nutrients, and water movement [41,42]. In addition, the *Sargassum* is not only composed of *S. natans* and *S. fluitans*, but there are also microorganisms (bacteria, microalgae, and invertebrates), together with the remains of other contaminating compounds that have been trapped within its composition [43]. The TS and VS content in this study were 87.02% and 77.78%, respectively, and resulted in a VS/TS ratio of 0.89. Comparing these results with other brown macroalgae, the VS/TS ratio of this study is high. The VS/TS ratio of brown macroalgae (*S. latissima*) was 0.83 [44] and Thompson et al. determined that the VS/TS ratio of pelagic *Sargassum* was 0.47 [11]; Milledege et al. found similar results to that reported by Thompson, the VS/TS relationship for a mixture of pelagic *Sargassum* was 0.53 [10]. Previous studies have reported optimum conditions for biogas production at a VS/TS ratio of 0.70 [45]; since the ratio of the present study is above 0.7, the *Sargassum* spp. collected in the Mexican Caribbean is a good option for anaerobic digestion.

### 4.2. Elemental Analysis

Table 2 shows the elemental analysis of this study. The ultimate analysis presented 33.84% of carbon; this value is higher than the result found by Milledge et al. with a composition of 27.41% to 29.23% for different *Sargassum* samples collected in Turks and Caicos [10]. The nitrogen content of the present study was 1.39%; this value is close to that reported in the literature for pelagic *Sargassum* collected in Turks and Caicos, which was 1.57% to 1.71% [10], and the pelagic *Sargassum* collected in Barbados at 1.21% [46].

This marine biomass has a C:N ratio of 24:1 which lies within the ideal C:N range of 20–35:1 for optimum microbial digestion and fermentation [47]. Compared with other brown algae, this result is very similar to the C:N ratio reported by Thompson, which was 21.67 [46]. A C:N ratio above the optimum causes methanogens to rapidly consume nitrogen to meet their protein needs, but they will stop consuming the remaining carbon in the substrate, reducing biogas production [48]. If the ratio is below the optimum, this indicates that the substrate is made up mainly of proteins and is rich in nitrogen, so the ammonia content will increase, inhibiting anaerobic digestion due to the change in pH in the digester that results in a toxic effect of the methanogenic stage [47]. Therefore, the C:N ratio found in the pelagic *Sargassum* collected during the autumn in this study is adequate for anaerobic digestion.

Based on the elemental analysis of pelagic *Sargassum,* the theoretical methane potential of this marine biomass was 405 ± 18.68 L CH_4_/kg VS, suggesting that pelagic *Sargassum* is a rich feedstock for mono-digestion and biomethane production. Compared with other macroalgae, this result was below that of *Ascophyllum nodosum*, *L. digitata*, *S. latissima*, and *Ulva lactuca*, which resulted in 488, 479, 422, and 465 L CH_4_/kg VS, respectively [49]. The differences are due to macroalgae being collected in different regions and seasons of the year, and being of different species [50]. Even when comparing the same species and the same collection region but collecting at different times of the year, the yield varies. This was described by Maneein et al., where the *Sargassum muticum* collected in the spring and summer present a theoretical yield of 397 to 463.8 L CH_4_/kg VS, respectively [50]. The theoretical yield of *Sargassum* spp. collected in Conset Bay, Barbados (142.84 L CH_4_/kg VS) [11] is below that reported in the present study (405 ± 18.68 L CH_4_/kg VS), and when compared with the *Sargassum* spp. collected in Turks and Caicos (496 L CH_4_/kg VS) [10] the theoretical yield of the present study turned out to be lower. The samples of *Sargassum* spp. collected in the Mexican Caribbean during the summer of 2019 determined that the theoretical potential of *Sargassum* resulted in 839.65 L CH_4_/kg VS [51]; this value above the present study may be due to the time and area in which it was collected [50]. Finally, when compared with the ligno-cellulosic biomass, it was observed that the *Sargassum* yield was above that obtained from wheat straw (232 L CH_4_/kg VS), corn stalk (206 L CH_4_/kg VS), sorghum (242 L CH_4_/kg VS), and barley straw (229 L CH_4_/kg SV) and are among the yields obtained from organic waste such as feed residues, sewage sludge, and animal waste (200 to 500 L CH_4_/kg VS) [45,52,53].

### 4.3. BMP

#### 4.3.1. Physical Pretreatment

The physical pretreatments C and CC result in biochemical methane potential values of 224.19 ± 9.45 and 152.89 ± 2.00 L CH_4_/kg VS (46.44% ± 1.69 and 48.86% ± 3.41 concentration of methane in the biogas). Table 4 shows the comparative yields reported. The vast majority of studies carried out anaerobic digestion under mesophilic conditions. The studies used different inoculums; in each of them, a blank was evaluated to correct the results obtained, and the methane accumulation reported is only from the macroalgae. The biochemical methane potential values of the literature are between 92 L CH_4_/kg VS [14] and 422 L CH_4_/kg VS [15], similar to those obtained in the present study. Milledge et al. determined a biodegradability of 17% to 39% for pelagic *Sargassum* samples when washed and cut [10]. Oliveira et al. determined that physical pretreatments increased biodegradability to 52% when compared to *Sargassum* without pretreatment [54]. Compared with these studies, the present work resulted in biodegradability of 55% for C and 38% for CC, close to the values reported by the literature. Regarding the increase in biogas production, the present study did not make a comparison with *Sargassum* without previous pretreatment, as described by other authors, for example, Nielsen and Heiske determined that *U. lactuca* resulted in a significant increase of 68% in methane from 152 to 255 L CH_4_/kg VS when compared with the macroalgae without pretreatment [44]; Yuhendra et al. concluded that physical pretreatment carried out on *Sargassum fulvellum* with particle sizes of 0.075–0.85 mm resulted in an optimal pretreatment that improved methane yields by 52.34% [22], while Tedesco et al. found a 52% increase in biogas production and 53% methane concentration when physical pretreatment (*L. digitata*) was performed and compared with the substrate without pretreatment [18]. In this study, the methane concentration in the biogas turned out to be below that reported by Tedesco et al. and the methane obtained from biogas is not the optimal 60–70% required [55,56] to be used in equipment such as electric power generators, however, physical pretreatments are important because the increase in the surface area and release of the sugars are necessary for anaerobic digestion [12], therefore, this pretreatment is necessary to carry out anaerobic digestion and must be accompanied by another pretreatment to increase the biodegradability of the biomass.

#### 4.3.2. Chemical Pretreatments

Physical pretreatment was carried out first, then chemical pretreatment with hydrogen peroxide at a low concentration (solution at 2.5% *v*/*v*) was performed to obtain yields of 230.82 ± 11.65 and 240.32 ± 3.04 L CH_4_/kg VS (51.55% ± 3.77 and 51.11% ± 0.56 concentration of methane in the biogas) for P and PC pretreatments, respectively. Comparing the pretreatments P and PC with the physical pretreatments (C and CC) resulted in an increased methane production of 3% and 57%, respectively. In this study, a slight improvement in methane production can be observed for P pretreatment, however, for PC pretreatment, the increase is greater. Biodegradability (57% and 59%) turned out to be higher than those obtained from physical pretreatment (C and CC). The results obtained were compared with other studies carried out on different macroalgae as shown in Table 4; the yields obtained with HCl pretreatment were 52 to 312 L CH_4_/kg VS [20,22,58,59]. P and PC are within the reported yields; however, some authors reported a decrease in methane production due to the release of inhibitors such as furfural, organic acids, and phenolic compounds [23]. The use of an HCl-like pretreatment of *S. fulvellum* resulted in a decrease of 8% in biogas production [22]; the same occurred with Ulva sp. (HCl), i.e., methane reduction was 42% when compared to macroalgae without pretreatment [20]. Barbot et al. determined that yields are affected according to acid concentration; for example, a concentration of 0.05 M experienced a 24% reduction, however, when the HCl concentration was 0.2 M, there was a slight increase of 3% when compared to a substrate without pretreatment before anaerobic digestion [58]. The use of lactic acid for *L. digitata* and *S. latissima* resulted in a 37% decrease in methane production [29]. Table 4 shows that the yields of alkaline pretreatments (NaOH) vary from 148 to 365 L CH_4_/kg VS for different macroalgae [20,21,22], with P and PC within the reported yields, as well as the use of acids in the use of NaOH, resulting in a 19% reduction in methane yield for *S. fulvellum* [22].

As shown in Table 4, there are no studies currently using hydrogen peroxide as a pretreatment in macroalgae anaerobic digestion, however, the production of bioethanol from *Ulva prolifera* used hydrogen peroxide as a pretreatment at a concentration of 0.5% *v*/*v*; this increased the presence of reducing sugars to 7.1 g/L and improved the efficiency of enzymatic hydrolysis by 31.4% [62]. The success of pretreatment with hydrogen peroxide is mainly due to its oxidative action since the derived radicals (OH and O_2_) depolymerize lignin by attacking lignin side chains and fragmenting its macrostructure into a number of low-molecular-weight compounds [63]. Even though pretreatment P resulted in a slight improvement regarding the physical pretreatment, the pretreatment PC increased methane production by 57.18%; in the bibliography, it was observed that the use of peroxide pretreatment has been successful in the pretreatment of terrestrial plants since it allows the release and increase in reducing sugars, in addition to being carried out under moderate pressure and temperature conditions [64]. In this study, the pretreatment was carried out at room temperature, so high-energy requirements were not necessary, and the agitation was only carried out for 3 h, unlike the acid treatments that require temperatures of 150 °C to 80 °C [20,58]; by not using acids or other agents under severe conditions, the use of hydrogen peroxide resulted in the formation of minor inhibitors [64] and since it does not require high-energy demands, it is a good option for *Sargassum* pretreatment.

#### 4.3.3. Enzymatic Pretreatment

The yields of pretreatments E and EC were 172.57 ± 0.56 and 179.56 ± 0.50 L CH_4_/kg VS with an 84.56% ± 0.15 and 81.49% ± 1.02 concentration of methane in the biogas, respectively. Comparing the yields with other studies (Table 4), it was observed that the methane yields were within those reported in the literature, from 49 L CH_4_/kg VS [14] to 232 L CH_4_/kg VS [20]. However, the yields reported in Table 4 show the use of pretreatments under different conditions and substrates, however, only in the study carried out by Tapia-Tussell et al. were the enzymatic pretreatment (enzymes from *Trametes hirsuta* BM-2) and substrate (*Sargassum* consortium in the Mexican Caribbean) similar [9]. In the case of fungi, the present study was based on the methodology by Tapia-Tusell et al. with differences such as the culture medium in which the fungus was isolated and the cultivated time (4 to 5 days). Another difference was the time the fungus remained in the YMPG medium (7 days) at pH 5. In addition, the *Sargassum* used in Tapia-Tussell was collected in Progreso (Yucatán, Mexico), whose composition was affected by the location of origin and varies from season to season [40], i.e., the chemical composition, growth, and pigmentation of *Sargassum* are significantly affected by conditions such as light, temperature, salinity, available nutrients, and water movement [41,42]. The study carried out by Tapia-Tussel et al. resulted in 104 L CH_4_/kg VS [9], below the yield obtained in this study of 172.57 ±0.56 (L CH_4_/kg VS).

Comparing the results obtained from E and EC with the physical pretreatment (C and CC), the methane yield for E and EC decreased by 23% and 17.44%, and compared with the chemical pretreatments (P and PC), E and EC have a lower yield (Table 4); the same occurs with biodegradability (for E and EC 42% and 44%, respectively). The methane production is affected by the transfer of electrons from the enzyme laccase to the substrate [65,66]; the oxidation of the phenylpropanoid units that lignin is made of, such as p-hydroxyphenyl [67], is involved in the formation of phenoms radicals [68] such as furfural, hydroxy-methylfurfural, or phenolic and/or aromatic compounds that inhibit anaerobic digestion [69,70]. Yuhendra et al. obtained a 62% decrease in biogas yield when using an enzymatic pretreatment [22]. Although the results obtained with the enzymes were not as favorable when compared to the physical and chemical pretreatments, this pretreatment is advantageous because *T. hirsuta* Bm-2 is a native fungus and does not involve the purchase of commonly expensive enzymes, in addition, enzymes are extracellular so they do not require the use of solvents or any extraction method, unlike other enzymes [71], and the culture medium was obtained at 35 °C, so the energy requirements are not high, contributing to the care of the environment.

#### 4.3.4. Combined Pretreatments

The combined pretreatments consisted of a physical pretreatment, followed by 2.5% hydrogen peroxide, and finally, the use of enzymes (PE and PEC); in addition, a combined pretreatment with a physical, chemical, and thermal pretreatment was evaluated before carrying out the enzymatic hydrolysis. The evaluated pretreatments PE, PEC, PTE, and PTEC presented a methane yield of 387.64 ± 1.41; 385.73 ± 4.76; 364.95.64 ± 8.18; and 318.06 ± 10.24 L CH_4_/kg VS, respectively (86.41% ± 0.04; 76.04% ± 0.80; 87.69% ± 1.49; and 85.26% ± 0.10 concentration of methane in the biogas, respectively), the biodegradability index was 95%; 95%; 88%; and 78%, respectively, as shown in Table 3. The results obtained from these pretreatments are very favorable; the yields obtained are very close to the theoretical (405 ± 18.68 L CH_4_/kg VS), completely degrading the *Sargassum,* leading to a percentage of methane obtained from the biogas above the optimum (60–70%) [55,56]. Comparing these pretreatments with physical pretreatment C yields methane increases of 73% and 63% for PE and PTE, respectively. Compared with the chemical treatment (P and PC), the increase in methane production was 68%; 60.5%; 58%; and 32% for PE, PEC, PTE, and PTEC, respectively. The combined pretreatment (enzymes and acid) applied to *L. digitata* and *S. latissima* resulted in a yield of 161 L CH_4_/kg VS [29], another combined pretreatment (pressure followed by an enzyme treatment) applied to *F. vesiculosus* resulted in an accumulation of 49 L CH_4_/kg VS [14]; the yields obtained for the *Sargassum* spp. collected in the Mexican Caribbean were above what is cited in the literature.

Although the four pretreatments presented better results, PTE and PTEC had a reduction in methane accumulation of 6% and 17% compared to PE and PEC because the temperature has a profound influence on inhibitor reaction kinetics, in addition, the reaction kinetics of lignin are similar to the degradation of reducing sugars [72]. Another disadvantage of the thermal pretreatment used in PTE and PTEC is the high-energy demands, for this reason, PTE and PTEC are discarded as a good pretreatment for the methanization of *Sargassum* spp. Therefore, PE and PEC accumulated a higher yield of methane; the success of these combined pretreatments was because chemical pretreatment with peroxide depolymerizes lignin [63], additionally, peroxide is not as severe as acids or other chemical agents that favor the formation of inhibitors from lignin [64]. When enzymatic pretreatment is carried out after the use of peroxide, the laccase oxidizes the phenolic compounds released, allowing greater degradation of lignin and phenolic inhibitors [66,73,74]. These pretreatments have an advantage in that the use of hydrogen peroxide at a low concentration (solution of 2.5%) can be carried out at room temperature without requiring additional energy demand, in addition to the use of an enzymatic extract without the use of solvents, which helps reduce the generation of pollutants and is favorable to the economy of the process for scaling in real conditions.

#### 4.3.5. DIET with Granular Activated Carbon (GAC)

Although the use of different conductive materials (GAC, biochar, nano-magnetite, and minerals) in the production of methane has been evaluated [31,32], this work has focused on GAC, mainly due to its high conductivity of 3,600 µS·cm^−1^; this conductivity is higher than magnetite (160 µS·cm^−1^) and biochar (5 µS·cm^−1^) [75]. GAC is the ideal conductive material due to its surface area, high conductivity, and economically favorable low cost [76,77].

The evaluated pretreatments with GAC showed that there are no significant changes in the accumulated yield of methane (analysis of variance ANOVA). PE and PEC pretreatments obtained the best yields with an average 86.14 ± 10% concentration of methane in the biogas, being above the optimum (60–70%) [55,56]. The same effect was determined by Cheng et al., who obtained a reduction of 40 to 45% in methane yields when adding biochar and GAC-like conductive material (a pig wastewater-like substrate). This effect is due to the GAC adsorption properties, this conductive material removed 11 to 17% of COD, in addition, the adsorption of organic compounds was observed [78]. The higher adsorption of volatile fatty acids from GAC has been observed with substrates whose volatile fatty acid (VFA) profile consists of hydrophobic compounds and long chains (above C5), for which a decreasing adsorption affinity was determined in the order of butyric, propionic, and acetic acid, which can cause a decrease in methane yields [79,80]. On the other hand, this can help keep VFAs below the inhibitory limits (less than 1.5 g/L) [81,82]; Valero et al. determined that the adsorption or promotion of DIET when a conductive material was used depended on the characteristics of the substrate; in the case of pot ale whiskey, the use of activated carbon did not increase anaerobic digestion due to the adsorption of organic matter, which in turn reduced the production of methane, while the powdered activated carbon (PAC) improved anaerobic digestion using brewery spent yeast as substrate and provided better resistance to inhibitory conditions with a yield of 699 L CH_4_/kg VS [34]. Conductive materials prevent the accumulation of volatile fatty acids, maintaining the optimal pH for biogas production (6.8 to 7.4) [83]. Shanmugam et al. used the GAC to adsorb inhibitors such as phenolic compounds and alter the concentration of COD before the anaerobic digestion begins, and, similar to other authors, the reduction in the concentration of methane was mainly due to the characteristics of the substrate and the adsorption properties of the conductive material [35]. Florentino et al.; 2019, found that the characteristics of the substrate can promote the adsorption of organic matter and correspond to a profile of COD and low free fatty acids, so when using GAC in the anaerobic digestion of blackwater at concentrations of 2.6 to 4.6 g COD/L (corresponding to 35 and 37% soluble COD), a reduction in soluble COD from 27 to 66% was obtained during the first 3 days, unlike when anaerobic digestion was carried out without GAC (increase in soluble COD of 60%). This demonstrated the adsorption of organic matter; regarding volatile fatty acids, the same behavior was observed when GAC was not used, acetate was 1277 mg/L, however, when GAC was added, acetate was 280 and 242 mg/L. However, at a concentration of 18.5 g COD/L, acetate was 1578 mg/L [84]. Although the use of GAC has been shown to promote DIET to increase methane production and quality, its use can also help improve pH and maintain redox potential within the range of methane formation (values below −250 mV) [34], in addition, its adsorption property could help reduce inhibitors [35]; however, the promotion of DIET depends on the concentration of GAC [85,86,87], as well as the addition of COD, that can favor the adsorption properties of GAC [84], so it is necessary to continue evaluating the concentration ratios and specific characteristics of each type of GAC, in anaerobic digestion tests when *Sargassum* spp. collected in the Mexican Caribbean was used as substrate.

## 5. Conclusions

The use of a conductive material such as GAC did not generate significant changes according to the analysis of variance performed (ANOVA) regarding performance and the concentration of methane. The main highlight of this research is that the best results were obtained from the combined pretreatments of 2.5% hydrogen peroxide, followed by an enzymatic pretreatment, resulting in a biodegradability of 95%, and an accumulated yield of 387 ± 3.09 L CH_4_/kg VS. However, it is necessary to continue evaluating the concentration of GAC and its characteristics when using *Sargassum* spp.

Likewise, the pelagic *Sargassum* used in this study is promising for bio-methane production in the Mexican Caribbean zone and solves the problem of its handling. Future research is needed to support these results to scale up continuous anaerobic reactors, in addition, techno-economic and environmental studies are necessary.

## Figures and Tables

**Table 1:** Moisture, total solids (TS), volatile solids (VS), and ash.

Parameter (%)	*Sargassum* spp.
Moisture%	12.98 ± 4.34
TS%	87.02 ± 4.34
VS%	77.78 ± 1.95
Ash	22.22 ± 1.95
VS/TS	0.89

**Table 2:** CHNS and theoretical methane potential (L CH4/kg VS).

Parameter (%)	*Sargassum* spp.
Ash%	22.22 ± 1.95
C%	33.84 ± 1.05
H%	4.71 ± 0.22
N%	1.39 ± 0.22
S%	1.21 ± 0.22
O%	36.62 ± 3.37
C:N	24.26

**Table 3:** Biodegradability index.

Pretreatment	Test	BI
PHYSICAL	C	0.55
	CC	0.38
CHEMICAL	P	0.57
	PC	0.59
ENZYMATIC	E	0.42
	EC	0.44
COMBINED	PE	0.95
	PEC	0.95
	PTE	0.88
	PTEC	0.78

BI = biodegradability index = BMP/TMP (potential/theoretical). C: physical pretreatment; CC: physical pretreatment with GAC; P: chemical pretreatment (peroxide 2.5%); PC: chemical pretreatment (peroxide 2.5%) with GAC; E: enzymatic pretreatment; EC: enzymatic pretreatment with GAC; PE: chemical and enzymatic pretreatment; PEC: chemical and enzymatic pretreatment with GAC; PTE: chemical, thermal and enzymatic pretreatment; and PTEC: chemical, thermal, and enzymatic pretreatment with GAC.

**Table 4:** Results of the different pretreatments evaluated in this study and its comparison with other results in the literature.

Pretreatment	Substrate	Operating Conditions	Pretreatment Characteristics	Yield (L CH_4_/kg VS)	% Methane	Literature
Physical	*Sargassum* spp. (C)	40 °C Inoculum: substrate: 2:1	Washed, dried (80 °C), and particle size > 1 mm	224.19 ±9.45	46.44% ± 1.69	This study
	*Sargassum* spp. (CC)			152.89 ± 2.00	48.86% ± 3.41	
	*Sargassum fulvellum*	38 °C	0.075–0.85 mm	350	44%	[22]
	*Laminaria* spp.	50 °C	Particle size 0.075 mm	229	52%	[18]
		40 °C	0.075 mm	210	51%	
		30 °C	0.075 mm	220		
	*Ulva lactuca*	55 °C	Wash and cut	271	-	[13]
	*S. latissima*	Inoculum:substrate: 1:1	Wash, dried (80 °C), and 2-3 mm	422	-	[15]
	*A. nodosum* (brown seaweed)	Diary slurry and grass silage (37 °C and I:S;2:1)	Wash and 4 mm	215-217	-	[57]
	*L. Digitata*	Diary slurry and grass silage (37 °C and I:S;2:1)	Wash and 4 mm	267	-	[17]
	*S. latissimi*			258		
	*F. vesiculosos*	37 °C	1000 bar 1 × 10 mm in the end (1 cm)	92	-	[14]
	*U. lactuca*	53 °C	Wash and macerated	255	-	[44]
Chemical	*Sargassum* spp. (P)	40 °C Inoculum: substrate: 2:1	Washed, dried (60 °C), and particle size > 1 mm+ peroxide	230.82 ±11.65	51.55% ± 3.77	This study
	*Sargassum* spp. (PC)			240.32 ±3.04	51.11% ± 0.56	
	*S. fulvellum*	38 °C	0.075–0.85 mm HCl 40 mL/L	312	40%	[22]
	*Ulva* sp.	35 °C Inoculum: substrate: 2:1	4% HCl a 150 °C	77	61%	[20]
	Mixture of red and green macroalgae	35–40 °C	5–20 mm 0.05 M HCl 80 °C	66	-	[58]
			5–20 mm 0.2 M HCl 80 °C 20 min	90	-	
	*F. vesiculosus*	37 °C	0.2 M HCl	52	-	[59]
	*L. digitata* and *S. latissima*	35 °C	1% lactic acid	161	-	[29]
	*Ulva* sp.	35 °C Inoculum: substrate: 2:1	4% NaOH 20 °C	148	57%	[20]
	*S. fulvellum*	38 °C	0.075–0.85 mm NaOH 10mL/L	282	43%	[22]
	*Palmaria palmata*	35 °C Inoculum: substrate: 2:1	0.04 g NaOH gTS−1 at 20 °C	365	-	[21]
Enzymatic	*Sargassum* spp. (E)	40 °C Inoculum: substrate: 2:1	Washed, dried (60 °C,) and particle size > 1 mm *T. hirsuta*	172.57 ± 0.56	84.56% ± 0.15	This study
	*Sargassum* spp. (EC)			179.56 ± 0.50	81.49% ± 1.02	
	*S. fulvellum*	38 °C	1 mL/L Viscamyl™ Flow cellulase enzyme	133.27	44%	[22]
	*pelagic Sargassum* spp.	38 °C	Enzymes from *T. hirsuta*	104	52%	[9]
	*Ulva* sp.	35 °C Inoculum: substrate: 2:1	Enzymes from *Aspergillus fumigatus*	153	58%	[20]
	*L. digitata* and *S. latissima*	35 °C	Cellulase	232		[29]
			Alginate lyase	225	-	
			Celluclast	72		
	*Rhizoclonium*	53 °C	Lipase	97		[60]
			Xylanase	77	-	
			α-amylase	79		
	*Oocystis* sp.	Inoculum: substrate:2:1	Commercial lacasse	100	-	[61]
			Lacasse from *T. versicolor*	144	-	
	*F. vesiculosos*	37 °C	Hemicellulase, pectinase, protease, and cellulase	49	-	[14]
Combined	*Sargassum* spp. (PE)		Washed, 60 °C particle size > 1 mm, peroxide and enzymes from *Trametes hirsuta*	387.64 ±1.41	86.41% ± 0.04	This study
	*Sargassum* spp. (PEC)			385.73 ± 4.76	76.04% ± 0.80	
	*Sargassum* spp. (PTE)		Washed, 60 °C particle size > 1 mm, thermal 120 °C, peroxide and Enzymes from *Trametes hirsuta*	364.95 ± 8.18	87.69% ± 1.49	
	*Sargassum* spp. (PTEC)			318.06 ± 10.24	85.26% ± 0.10	
	*L. digitata* and *S. latissima*	35 °C	Cellulase 1% lactic acid	161	-	[29]
	*F. vesiculosos*	37 °C	1000 bar + Hemicellulase, pectinase, protease, and cellulase	49	-	[14]
	*F. vesiculosus*	37 °C	80 °C and 0.2 M HCl	116	-	[59]

## Data Availability

Not applicable.
